# Osteoporosis: the current status of mesenchymal stem cell-based therapy

**DOI:** 10.1186/s11658-016-0013-1

**Published:** 2016-08-12

**Authors:** Jitrada Phetfong, Tanwarat Sanvoranart, Kuneerat Nartprayut, Natakarn Nimsanor, Kanokwan Seenprachawong, Virapong Prachayasittikul, Aungkura Supokawej

**Affiliations:** 1 Department of Clinical Microscopy, Faculty of Medical Technology, Mahidol University, Phuttamonthon, Salaya, Nakhon Pathom 73170 Thailand; 2 Department of Clinical Microbiology and Applied Technology, Faculty of Medical Technology, Mahidol University, Phuttamonthon, Salaya, Nakhon Pathom 73170 Thailand

**Keywords:** Osteoporosis, Cell therapy, Mesenchymal stem cells, MSCs, Stem cells

## Abstract

Osteoporosis, or bone loss, is a progressive, systemic skeletal disease that affects millions of people worldwide. Osteoporosis is generally age related, and it is underdiagnosed because it remains asymptomatic for several years until the development of fractures that confine daily life activities, particularly in elderly people. Most patients with osteoporotic fractures become bedridden and are in a life-threatening state. The consequences of fracture can be devastating, leading to substantial morbidity and mortality of the patients. The normal physiologic process of bone remodeling involves a balance between bone resorption and bone formation during early adulthood. In osteoporosis, this process becomes imbalanced, resulting in gradual losses of bone mass and density due to enhanced bone resorption and/or inadequate bone formation. Several growth factors underlying age-related osteoporosis and their signaling pathways have been identified, such as osteoprotegerin (OPG)/receptor activator of nuclear factor B (RANK)/RANK ligand (RANKL), bone morphogenetic protein (BMP), wingless-type MMTV integration site family (Wnt) proteins and signaling through parathyroid hormone receptors. In addition, the pathogenesis of osteoporosis has been connected to genetics. The current treatment of osteoporosis predominantly consists of antiresorptive and anabolic agents; however, the serious adverse effects of using these drugs are of concern. Cell-based replacement therapy via the use of mesenchymal stem cells (MSCs) may become one of the strategies for osteoporosis treatment in the future.

## Background

Osteoporosis, a bone disease involving the appearance of porous bone, is characterized by low bone mass and microarchitectural deterioration of bone tissues, leading to reduced bone strength and a consequent increase in fracture risk [1, 2]. Osteoporosis is increasingly recognized as a major public health concern that affects more than 200 million people worldwide and causes more than 8.9 million fractures, and mainly hip fractures, per year [3]. The incidence of osteoporosis has dramatically risen because the life expectancy of the population has been increasing in every geographic region [4]. The consequences of osteoporosis are significant, as is the financial burden, estimated at approximately US $17 billion for more than 2 million fractures in the US [5]. The prevention of this disease and its associated fractures is considered essential to health maintenance, quality of life, and independence in the elderly population.

According to the World Health Organization (WHO) criteria, osteoporosis is defined as having a bone mineral density (BMD) value that is 2.5 standard deviations or more (T-score ≤ -2.5) below the average value for young healthy women, as measured by dual-energy x-ray absorptiometry (DXA), which is the most validated technique (i.e., the gold standard) [1, 2]. A low BMD not only is a major risk factor for fractures but also is an independent risk factor for death [6]. BMD testing of the hip and spine is required for a densitometric diagnosis of osteoporosis. Measurements of bone strength other than bone density at these sites may predict fracture risk but cannot be used to diagnose osteoporosis [7]. BMD remains the best tool to assess fracture risk, but it cannot predict the fracture risk in certain cases, particularly in type 2 diabetes patients, who usually have a higher BMD and an increased fracture risk [8]. The majority of osteoporotic fractures occur in individuals with a BMD level within the osteopenic range (-2.5 < T-score < -1) or even with normal BMD levels [9]. The Fracture Risk Assessment (FRAX) tool integrates BMD and clinical risk factors such as age, gender, the history of fracture, the parental history of hip fracture, current smoking, excessive alcohol intake, rheumatoid arthritis, glucocorticoid use, and other causes of secondary osteoporosis. FRAX has been shown to be a more applicable prediction tool to estimate the risk and probability of fracture in an individual over the next 10 years. FRAX can also provide general clinical guidance for treatment decisions [10].

Over the past few decades, there have been great advances in understanding of the physiologic process of bone remodeling, together with associated pathologic conditions. The underlying pathogenesis of osteoporosis involves an imbalance of bone homeostasis that results from many causes, such as hormone deficiency, genetic disorders, use of certain medications, and medical conditions. Osteoporosis is characterized by low bone mass and density, which lead to an increased fracture risk. The aims of treatment for osteoporosis are to reduce bone loss and maintain bone density, especially in patients who have fractures or a high risk of fractures. Many therapeutic drugs for treating osteoporosis are available in the market, most of which have relied on bone resorption inhibition, such as bisphosphonate, and several drugs are being developed for treatment in the future. However, controversies have confounded the treatment of osteoporosis. Thus, new treatments based on the promotion of bone regeneration or alternative cell-based therapy for osteoporosis patients are expected to be investigated. Stem cells are expected to have great therapeutic potential, particularly in regenerative medicine. Specifically, stem cells could be a promising cell source for cell-based therapy for osteoporosis. In the present review, the current understanding of mesenchymal stem cells (MSCs) and their roles in osteoporosis, the genetics and transcriptional regulation involved in the pathogenesis of osteoporosis, the signaling pathways associated with osteoporosis and trends in using stem cells as cell-based therapy for osteoporosis is summarized.

### Pathogenesis

#### Imbalance of bone homeostasis

Bone formation generally comprises three basic steps: synthesis of extracellular protein matrix (osteoid) by osteoblasts; matrix mineralization by coating the protein matrix with a layer of mineral, and predominantly calcium phosphate in the form of crystals of hydroxyapatite; and bone remodeling, which is a process that occurs throughout human life. Bone remodeling is essential to maintain the integrity of the skeleton and serves as storage for mineral homeostasis [11]. Via an interactive process called coupling, this process is balanced by the functions of bone-resorbing osteoclasts and bone-forming osteoblasts in early adulthood. When the bone loses its mineral content and density and develops osteopenia, this culminates in osteoporosis, which is associated with a risk of bone fractures [11, 12].

Osteoporosis is normally related to increasing age, consistent with the fact that most of the older population is affected by this condition. It has been shown that genetics could be another explanation for the pathogenesis of osteoporosis. The results of laboratory studies have indicated that osteoporosis is caused by an imbalance of the coupling interactive process, with increased bone resorption relative to bone formation. In this regard, the imbalance is a consequence of changes at the cellular level, by which osteoclast development is enhanced but osteoblast differentiation is insufficient because of impaired activity and enhanced apoptosis [13, 14].

### Underlying transcriptional regulation and genetics

Although MSCs have the ability to undergo multipotent differentiation, cell fate determination and differentiation toward either osteoblasts or adipocytes are well regulated by lineage-specific transcription factors such as runt-related transcription factor 2 (Runx2) and osterix (Osx) for osteoblasts and peroxisome proliferator-activated receptor gamma (PPARγ) for adipocytes, suggesting an inverse correlation between osteogenesis and adipogenesis [15–19]. During these processes, intrinsic (genetic) and/or environmental (local and/or systemic) conditions interplay to specify cell fate toward one of the possible lineages. Several lines of evidence have demonstrated that osteoporotic MSCs have defects in intrinsic signals that cause functional alterations, leading to poor osteogenic differentiation capacity and favoring increased adipogenesis [13, 20]. A recent study of microarray analyses demonstrated that MSCs from elderly patients with primary osteoporosis have a distinct transcriptome compared with control MSCs and elderly donor non-osteoporotic MSCs, as shown by enhanced mRNA expression of osteoporosis-associated genes (*RUNX2,* lipoprotein receptor-related protein 5; *LRP5,* collagen type 1 alpha1; *COL1A1*), genes involved in osteoclastogenesis (*CSF1, PTH1R*), and genes coding for inhibitors of wingless-type MMTV integration site family (Wnt) and bone morphogenetic protein (BMP) signaling, indicating intrinsic deficiencies in self-renewal and differentiation potential in osteoporotic MSCs [21]. Interestingly, transcriptional alterations may reflect epigenetic changes as part of the process of age-related osteoporosis [21].

Nevertheless, the regulatory mechanisms underlying the pathogenesis of osteoporosis have been linked to genetics. Several approaches, including linkage analysis in families, animal studies, candidate gene association studies, and genome-wide association studies (GWAS), have been used to identify the genes responsible for osteoporosis [22]. Linkage analysis is the classical approach that is used to study BMD variation [23, 24]. Linkage studies in animals such as mice, rats and primates provide another way to identify genes that regulate bone density and other phenotypes relevant to osteoporosis. The *Alox15* gene has been found to regulate bone density in mice, and this finding was confirmed in *Alox15*-knockout mice showing increased BMD [25]. Candidate gene association studies have been widely used in the field of osteoporosis, analyzing polymorphic variants in candidate genes and relating them to the carriage of a specific allele or haplotype. Candidate genes such as sclerostin (*SOST*), *COL1A1*, *ESR1*, *LRP5*, *TGF-β1* and *VDR* have been extensively investigated on a large scale [26–40]. Due to advances in genotyping technologies, GWAS have been applied to study osteoporosis, and large numbers of single-nucleotide polymorphisms (SNPs) have been identified. A GWAS by Richards et al. reported the identification of SNPs that are significantly associated with decreased BMD and increased risks of osteoporotic fractures and osteoporosis when they are located near the *TNFRSF11B* (osteoprotegerin or OPG) and *LRP5* genes [41]. Another study, by Styrkarsdottir et al., used an extended GWAS to identify four new genome-wide significant loci; this loci were near the *SOST* gene at 17q21, the *MARK3* gene at 14q32, the *SP7* gene at 12q13 and the *TNFRSF11A* (receptor activator of nuclear factor kB or RANK) gene at 18q21 and were associated with the heritability of BMD [42]. However, genetic studies of osteoporosis-susceptibility genes need to be further explored.

### Signaling pathways associated with osteoporosis

Over several decades, signaling pathways in bone homeostasis have been extensively studied. Dysregulation of these signaling pathways is associated with bone diseases, including osteoporosis. Major signaling pathways that govern the bone regenerative process are OPG/RANK/RANK ligand (RANKL), Wnt, and BMP signaling.

**Fig. 1:**
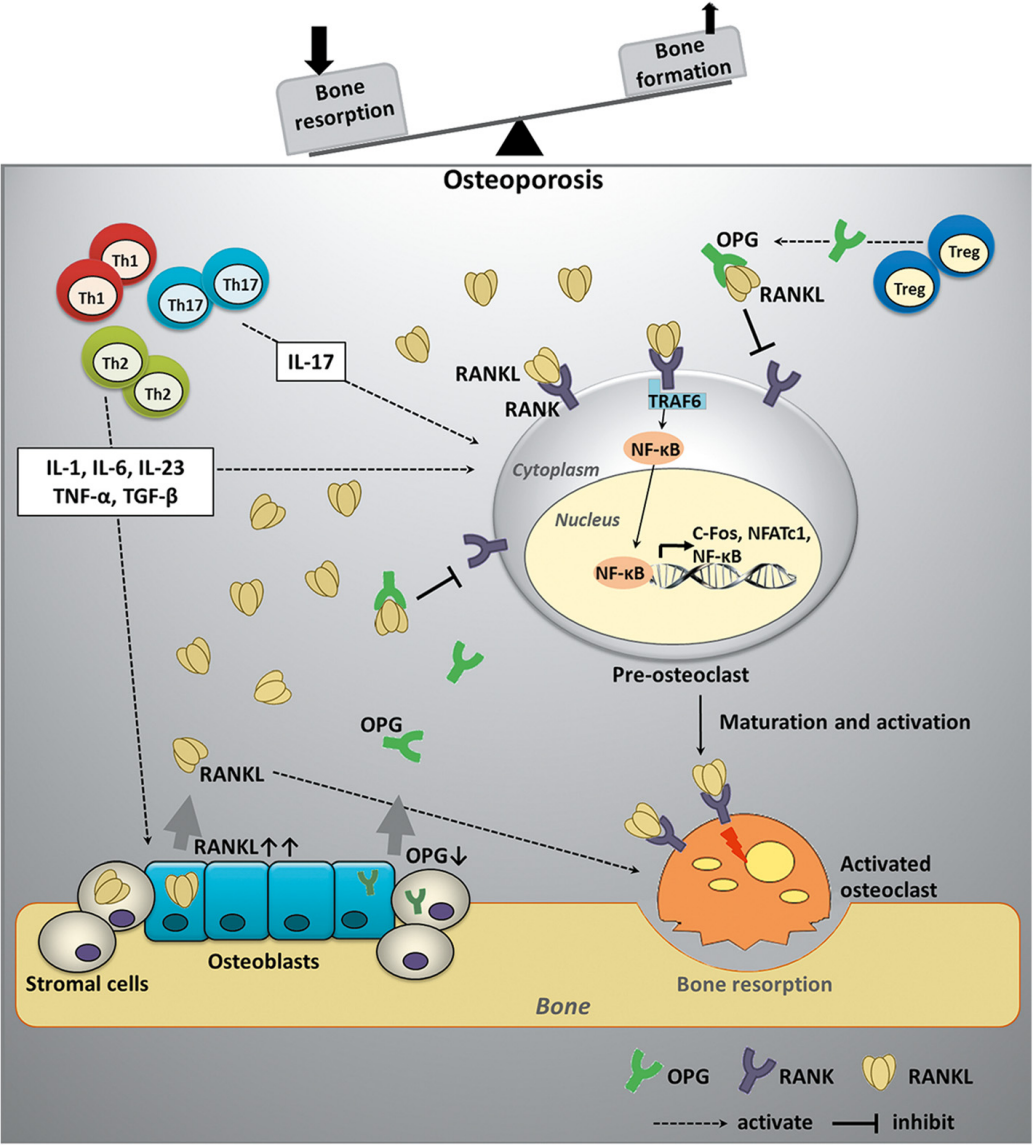
Bone homeostasis regulation by OPG/RANK/RANKL system. RANKL which secreted by activated T cells functions as an osteoclast-activating factor by binding to its receptor, RANK, which is expressed on preosteoclasts. RANKL-RANK binding induces the activation of several transcription factors in preosteoclasts and initiates several downstream signaling pathways that drive osteoclast differentiation and maturation. OPG which secreted by osteoblasts, bone marrow stromal cells, and T reg cells acts as a soluble receptor that can bind to RANKL and subsequently prevents RANKL-RANK binding. Under physiologic conditions, OPG/RANKL is in equilibrium and preserves bone homeostasis. Under osteoporotic conditions, RANKL is upregulated, which is associated with downregulation of OPG. Several proinflammatory cytokines are secreted from T helper cells (Th1/Th2/Th17) stimulating and upregulating RANKL expression and mediating osteoclast formation and activity, which are linked to increased bone resorption

Bone homeostasis is maintained by the balanced function of osteoblasts and osteoclasts. The key regulators involved in this balancing process, equilibrating between bone formation and bone resorption, have been extensively explored. The OPG/RANK/RANKL system is one of the most important signaling pathways in bone metabolism (Fig. 1). Dysregulation of the OPG/RANK/RANKL system has been reported in osteoporosis. OPG, recently designated as TNFRSF11B and serving as a member of the tumor necrosis factor (TNF) receptor family, was first identified as a crucial component that is secreted by osteoblasts; bone marrow stromal cells [43]; and other cells, such as regulatory T (T reg) cells [44]. OPG protects the skeleton from excessive bone resorption by acting as a soluble decoy receptor that can bind to RANKL [45]. The binding of OPG and RANKL subsequently prevents RANKL from binding to its receptor, RANK [43]. The overexpression of the gene encoding OPG results in the development of high bone mass and reduced osteoclast numbers and activity [46]. OPG-deficient mice demonstrate osteoporosis, with an excessive number of osteoclasts [47, 48]. RANKL functions as an osteoclast-activating factor secreted by activated T cells and represents a potent molecule that binds to RANK, which is expressed on osteoclast precursors, known as preosteoclasts [49]. RANKL-RANK binding drives osteoclast differentiation and maturation. The activation of RANK through the binding of RANKL induces the activation of transcription factors such as c-fos, NFAT, and nuclear factor kappa B (NF-kB) in preosteoclasts and initiates several downstream signaling pathways, and especially the NF-kB pathway [50, 51]. RANKL-deficient mice exhibit osteopetrotic bones, or thickened bones, due to a defect in osteoclast development [52]. Moreover, RANKL relies on the presence of macrophage colony-stimulating factor (M-CSF), which is a cofactor for RANKL/RANK-mediated osteoclastogenesis [53]. However, experimental data revealed that RANKL alone could stimulate bone resorption in mice lacking M-CSF [54]. In contrast, M-CSF alone is insufficient to activate osteoclasts [55]. Therefore, RANKL plays a crucial role in osteoclastogenesis, and this phenomenon is required for bone resorption. Under physiologic conditions, OPG/RANKL is in equilibrium and preserves bone homeostasis. The OPG/RANKL ratio is an important factor to use to determine bone mass and skeletal integrity [56, 57]. Under osteoporotic conditions, RANKL is upregulated, which is associated with downregulation of OPG [58]. Moreover, several cytokines are elevated, and particularly TNF-α, IL-1, IL-4 and IL-6, in osteoporosis [59]. These proinflammatory cytokines modulate the RANKL/RANK ratio by stimulating and upregulating RANKL expression on T cells. Interestingly, this emerging role of the OPG/RANK/RANKL system not only is relevant to bone biology but also has been discovered beyond the immunological system. The cross-regulation between bone and immune cells is considered as a bone immunological niche [60]. Considering bone resorption, data in the literature have revealed that impairment of T cell subpopulations and their proinflammatory cytokine patterns are implicated in the pathogenesis of osteoporosis. At the bone tissue level, Th1 and Th2 cells play a role through their secreted cytokines, including RANKL, mediating osteoclast formation and activity, which are linked to bone resorption [61]. Furthermore, Th17 cells, a distinct lineage of proinflammatory T helper cells, were more recently identified as a potent T cell subpopulation that has a role in bone destruction [62]. Th17 cells have been found to increase in number in many bone diseases, and particularly osteoporosis [63]. Th17 cells produce IL-17, which functions in mediating osteoclast differentiation [63, 64]. It has been shown that Th17 cells also produce RANKL, directly contributing to bone loss [62]. Additionally, the Th17 population in the bone marrow and peripheral blood is large in estrogen-deficient osteoporosis [65]. Collectively, Th1/Th2/Th17 cells and their cytokines might play a key role as potent pro-osteoclastogenic mediators underlying the pathogenesis of osteoporotic development.

Wnts are secreted glycoproteins that, when bound to their cognate receptors, can stimulate intracellular signaling cascades that play important roles in cell developmental processes, including osteogenesis [66, 67]. The binding of Wnt ligand to Frizzled receptor and the LRP6 coreceptors to form a complex stimulates the canonical Wnt/β-catenin pathway, whereas binding to the ROR2/RYK coreceptors stimulates non-canonical Wnt signaling [68]. In fact, signaling induced by Wnt/β-catenin is well established and generally plays a role in osteoblastogenesis by promoting the commitment and differentiation of MSCs toward the osteoblast lineage, which in turn suppresses adipogenesis through the inhibition of PPARγ-induced genes [66, 69]. Wnt/β-catenin signaling plays a role in osteoblast maturation and indirectly reduces osteoclastogenesis by stimulating the secretion of OPG, a natural inhibitor of RANKL [70, 71]. Considering the components of Wnt signaling, humans and mice with altered expression of LRP5 and Wnt10b have alterations in bone mass [72, 73]. Loss-of-function *LRP5* mutation causes abnormality in bone formation [72]. A genetic study found that *WNT10B* polymorphisms have an impact on low bone mass and osteoporosis risk [74]. As previously stated, Wnt10b seems to be the most positive modulator of bone regeneration and homeostasis. Supporting these findings, a decreased number and decreased function of osteoblasts have been found in *Wnt10b* ^-/-^ knockout mice, coupled with a 30 % reduction in bone volume and BMD [75, 76]. Stevens et al. have found that heterozygous *Wnt10b* ^*+/-*^ mice showed a significant reduction in trabecular bone at 6 months of age, and both the number of bone marrow-derived MSCs and osteoblast differentiation were affected [76]. In another study, signaling through Wnt2, Wnt3 or Wnt3a induced proliferation and maintained the self-renewal of MSCs, whereas Wnt5a, Wnt5b or Wnt11 supported osteogenesis [77, 78]. *β*-catenin deficiency arrests osteoblast development at an early stage in mesenchymal osteoblastic precursors and impairs the maturation and mineralization of committed osteoblasts [67, 79]. Rodríguez et al. reported that osteoporotic MSCs had a diminished proliferation rate as well as decreased mRNA expression of Wnt signaling and the downstream components *GSK-3β, LRP6* and *OSX* [28, 80]. In addition, dickkopf-1 (DKK-1) and SOST are endogenous inhibitors of the canonical Wnt/β-catenin pathway that is specific to bone [70, 81]. Genes coding for these inhibitors show enhanced expression in osteoporotic MSCs in humans [35]. Clinically, the serum DKK-1 level has been found to be significantly higher in patients with low BMD and postmenopausal osteoporosis [82]. Several findings have revealed crosstalk between Wnt signaling and other signaling factors, such as BMPs. In particular, BMP-2 has a synergistic effect with Wnt ligands and β-catenin, inducing bone formation through Wnt/β-catenin signaling and downstream T cell factor/lymphoid enhancer factor (TCF/LEF) transcriptional activity [83, 84].

In addition to Wnt signaling, the BMPs, belonging to the transforming growth factor beta (TGF-β) superfamily, are responsible for numerous cell regulatory processes, including osteogenic differentiation and regulation of bone formation [85]. Upon binding of BMP ligand, signal transduction is initiated through the interaction between two serine-threonine kinase cell surface receptors (BMP receptors (BMPRs)). In particular, BMPR-IA and BMPR-IB are involved in MSC differentiation [86]. BMP-2, BMP-4, BMP-7, BMP-9, and BMP-13 are commonly studied in the context of MSC differentiation-related osteoblastogenesis and bone formation [87, 88]. Notably, BMP-2 promotes Runx2 expression in mesenchymal osteoprogenitors and also promotes Osx and distal-less homeobox 5 (Dlx5) expression in osteoblasts [89–93]. BMP-3 is an exception because it inhibits osteogenesis [94]. BMPs function as both autocrine and paracrine factors, and their synthesis is induced by BMPs themselves via local feedback mechanisms. Evidence has shown that MSCs from osteoporosis patients are impaired in function and this alteration is associated with BMP signaling [95, 96]. However, BMP antagonists have been described, including noggin (NOG) and gremlin (GREM). Overexpression of NOG, as shown in transgenic mouse studies, results in decreased BMD because of increased inhibition of bone formation [97, 98]. SNPs in the *NOG* gene are associated with osteoporosis-related phenotypes in humans [99]. GREM is detectable in the skeleton, and its overexpression causes osteopenia and fractures [100]. Genetic variants of GREM2 are associated with BMD, and *GREM2* is considered a susceptibility gene for osteoporosis [101].

### Osteoporosis treatments

Current options for the treatment of osteoporosis are predominantly drug-based agents that either inhibit bone resorption or directly stimulate bone generation to increase bone mass. Non-pharmacological treatments via calcium and vitamin D consumption have been given to patients who have a high risk of osteoporosis related to insufficient calcium and vitamin D intake and postmenopause [102, 103]. Pharmacological treatments are given to patients who are diagnosed with osteoporosis who have already had a fracture or who have a high risk of osteoporotic fracture or re-fracture. Bisphosphonates, which are synthetic compounds that decrease bone resorption by promoting osteoclast apoptosis [104, 105], are the most common medications prescribed as first-line drugs for osteoporosis treatment. Several bisphosphonates have been approved as drugs for the treatment of osteoporosis, including alendronate [106], ibandronate [107], risedronate [108], and zoledronate [109]. However, serious side effects, such as osteonecrosis of the jaw and atypical femoral fractures, have been described in patients under long-term bisphosphonate treatment [110, 111]. Although serious adverse events are rare for current antiresorptive compounds and do not represent a major concern, the development of drugs with higher efficacy in improvement of bone quality and prevention of fractures is still necessary. Other antiresorptive drugs that can serve as alternatives for osteoporosis treatment include denosumab, a RANKL inhibitor that blocks the main pathway involved in osteoclast formation and activation [112], and calcitonin, a naturally occurring peptide [113]. Hormone therapy, such as therapy with estrogen [114] and with selective estrogen receptor modulators (SERMs) acting as estrogen agonists, such as raloxifene [115], has been used in postmenopausal women to slow the bone breakdown process, maintain bone density, and reduce fracture risk. However, long-term side effects, and particularly the development of breast cancer, and risks of cardiovascular events and thromboembolism limit the use of estrogen and SERMs as treatment strategies for osteoporosis [116, 117].

In contrast with antiresorptive drugs, anabolic drugs that can increase bone formation, rather than preventing bone loss, are of interest to rebuild bone, increase bone strength, and reduce the risk of fractures in osteoporosis patients. To date, approved anabolic drugs have been limited to parathyroid hormone (PTH) and its analog, teriparatide (recombinant human PTH(1–34)), which are considered as treatments for patients with severe osteoporosis. Nevertheless, it was reported that administering a high dose of teriparatide for a long period increased the incidence of osteosarcoma in an animal study [118]. Although evidence of osteosarcoma has not been reported in patients taking teriparatide, treatment with teriparatide is not allowed beyond 2 years according to the FDA. One therapeutic drug, strontium ranelate, which is thought to have dual actions on bone metabolism, both increasing bone formation and decreasing bone resorption, represents as a potential agent for the treatment of postmenopausal osteoporosis to reduce the risk of vertebral and hip fractures [119]. Considering the costs and disadvantages of prolonged treatment with drugs and hormones in osteoporosis patients, cell therapy may be a good alternative candidate therapeutic strategy to treat osteoporosis in the future.

### Stem cell-based therapy for osteoporosis

Cell therapy has attracted considerable clinical attention for the treatment of various diseases for many decades. Stem cells are believed to be an ideal source of cells for cell replacement therapy for bone diseases due to their properties of self-renewal and plasticity, which can repair or regenerate damaged tissues. Candidate stem cell types include embryonic stem (ES) cells, induced pluripotent stem (iPS) cells and somatic stem cells such as MSCs. The use of ES and iPS cells is limited due to ethical issues and virus-based derivation methods [120]. It seems likely that the use of MSCs overcomes such limitations and is more practical in other disease models. In recent years, MSCs have become dramatically interesting for the treatment of osteoporosis. MSCs have the ability to self-renew and to grow into specific tissues, such as cartilage, bone, and adipose tissue. Human MSCs are defined by their phenotypic expression of CD105, CD73 and CD90; their absence of expression of hematopoietic markers such as CD45, CD34, and CD14; and their ability to differentiate into osteogenic, adipogenic and chondrogenic lineages under permissive conditions [121]. It has been reported that MSCs can avoid allogeneic rejection by being hypoimmunogenic, modulating the T cell phenotype, and creating an immunosuppressive locus [122]. Moreover, MSC-derived osteogenic cells show immunoprivileged and immunomodulatory properties similar to those of their parental MSCs [123]. With regard to the pathogenesis of osteoporosis, resulting in bone mass reduction, transplantation of MSCs might promote new bone formation and strengthen the bone, contributing to improvement of bone quality and prevention of fractures. After transplantation, MSCs contribute to bone formation by two possible mechanisms of action: (1) MSCs’ homing to a damaged site or pathologic area and then differentiating into bone-forming cells to repair the degenerated tissue and (2) MSCs’ acting in a paracrine manner by secreting certain growth factors that modify the environment and recruit resident cells to repair the degenerated tissue [124, 125].

### Sources of mesenchymal stem cells: advantages and disadvantages

#### Bone marrow-derived MSCs

Bone marrow is the most commonly used tissue source of adult MSCs. Bone marrow-derived MSCs (BM-MSCs) have been extensively studied in bone regeneration and repair due to their high efficiency in osteogenic differentiation. Studies in animal models have revealed that both allogeneic and autologous BM-MSC transplantation is applicable in the treatment of osteoporosis. Ichioka et al. demonstrated that normal allogeneic BM-MSCs could increase trabecular bone and attenuate the loss of BMD after being directly injected into the bone marrow cavity of an irradiated P6 substrain of senescence-accelerated mice (SAMP6), an osteoporotic mouse model that exhibits age-dependent inhibition of osteoblastogenesis and osteoclastogenesis along with enhanced adipogenesis [126]. A similar result was also observed in an ovariectomy (OVX)-induced rat model of osteoporosis after receipt of allogeneic BM-MSCs isolated from healthy rats [127]. Autologous BM-MSC transplantation was reported to improve bone formation and to strengthen osteoporotic bone in an OVX-induced rabbit model of osteoporosis [128] as well as in goats with long-term estrogen deficiency, mimicking the postmenopausal osteoporosis that occurs in humans [129]. However, use of autologous BM-MSCs for osteoporosis treatment in elderly patients is limited due to the age-related decline in the overall BM-MSC number [130]. Recently, use of autologous BM-MSCs for the treatment of osteoporosis has been performed in clinical trial study. Autologous BM-MSCs were collected 30 days before infusion, and the cells were cultured under GMP conditions to establish the dose range. In this study, the cells were subjected to the process of fucosylation before intravenous infusion into osteoporosis patients. However, this study is still in the process of recruiting participants and is thus not yet completed (ClinicalTrials.gov Identifier: NCT02566655).

### Adipose tissue-derived MSCs

Adipose tissue provides an attractive source of MSCs that has become increasingly popular in many stem cell applications. Adipose tissue-derived MSCs (AD-MSCs) are isolated from white adipose tissues via a minimally invasive approach and can be expanded and differentiated into classical mesenchymal lineages involved in adipogenesis, osteogenesis, and chondrogenesis [131, 132]. AD-MSCs are more easily isolated and more abundant and produce higher yields in terms of cell number compared with BM-MSCs [133]. However, the yield of AD-MSCs and their proliferative and differentiation capacities vary depending on the tissue harvesting site [134] and the age of the donor [135]. For application in cell therapy for osteoporosis, AD-MSCs were reported to function as an effective autologous cell-based approach for the treatment of osteoporosis. SAMP6 osteoporosis mice showed significant improvement in several trabecular bone parameters after a single intratibial transplantation of isogenic AD-MSCs [136]. A preclinical study of the in vivo function of human AD-MSCs by Cho et al. revealed that human AD-MSCs could prevent OVX-induced bone loss in nude mice over 8 weeks, even though there was no evidence of long-term engraftment of infused human AD-MSCs in the bone of recipient mice [137]. The effect of human AD-MSC therapy likely occurs in a paracrine manner by the secretion of various bone-related growth factors, e.g., hepatocyte growth factor, BMP-2, and RANKL, and extracellular matrix (ECM) proteins, e.g., fibronectin, which might promote osteogenic differentiation, bone remodeling and repair in the recipients [124]. Moreover, Xinhai et al. demonstrated that autologous AD-MSCs enhanced bone regeneration in OVX-induced rabbit models of osteoporosis due to not only their own osteogenic differentiation but also their promotion of osteogenesis and inhibition of adipogenesis by osteoporotic BM-MSCs through activation of BMP-2 and the BMPR-IB signaling pathway [125]. Recently, a clinical trial has studied the use of human AD-MSCs for the treatment of proximal humeral fractures in individuals over 50 years old, representing a model for fractures of osteoporotic bone. In this study, AD-MSCs were wrapped around hydroxyapatite microgranules embedded in a fibrin gel to allow cellularized composite graft augmentation. Clinical/radiological follow-up was performed after 6, 9 and 12 months, and functional assessment was performed after 6 weeks and 6 and 12 months using the Quick DASH score and the Constant score. Unfortunately, the study was terminated, and no results are available (ClinicalTrials.gov Identifier: NCT01532076).

### Perinatal-derived MSCs

Although BM- and AD-MSCs are effective sources, the therapeutic potential of these adult MSCs can be affected by the donor’s lifestyle and age. Perinatal tissues are alternative sources of MSCs that have attracted growing interest in bone regenerative medicine [138]. Not only are these cells younger than adult MSCs, but perinatal-derived MSCs also have the major advantage of an easy and noninvasive harvesting procedure without any risk to the donor. A comparative study of MSCs isolated from different perinatal tissue sites, including the umbilical cord, umbilical cord blood (UCB), amnion, and chorion, revealed that these tissues exhibit similar characteristics to BM-MSCs, including similar phenotypic features, growth properties, differentiation capacities, secretory protein profiles, and low immunogenic properties [138]. However, these stem cell sources are still limited by their low capacity to differentiate compared with BM- and AD-MSCs, and they have not been clearly examined in preclinical studies.

#### Placenta-derived MSCs

The placenta is an easily accessible source of perinatal MSCs that provides a high yield of MSCs. Placenta-derived MSCs (PL-MSCs) express common markers of MSCs and exhibit adipogenic, osteogenic, and neurogenic differentiation capacities [139]. Sanvoranart et al. demonstrated that PL-MSCs responded to bortezomib, a chemotherapeutic agent that improves osteolytic lesions in multiple myeloma, via enhancement of osteogenic differentiation, similarly to BM-MSCs [140]. This finding suggests the potential therapeutic application of PL-MSCs in osteopenia and osteoporosis patients.

#### Umbilical cord-derived MSCs

The umbilical cord contains various cell types, including vessels, connective tissues, and Wharton’s jelly. After isolation, these heterogeneous cells are observed to possess differential vimentin and cytokeratin expression in culture, but not variable capacities to differentiate into chondrogenic, adipogenic, and osteogenic lineages [141]. In vivo bone formation by umbilical cord-derived MSCs (UC-MSCs) was demonstrated by Diao et al., who loaded human UC-MSCs into scaffolds, implanted the scaffolds into BALB/c nude mice subcutaneously and found that the human UC-MSCs could efficiently form bone after implantation for 12 weeks [142].

#### Wharton’s jelly-derived MSCs

Wharton’s jelly is the mucoid connective tissue that surrounds the umbilical cord vein and that functions in the protection of the vasculature from pressure. Fibroblast-like cells were first isolated from Wharton’s jelly by McElreavey et al. in 1991 [143]. These fibroblast-like cells were characterized as MSCs due to their expression of MSC phenotypic markers and their capacities to differentiate into osteogenic, adipogenic, and chondrogenic lineages [144]. A comparative study of human derived-MSCs demonstrated that Wharton’s jelly-derived MSCs (WJ-MSCs) exhibited the strongest inhibitory effects on T cell proliferation and the weakest expression of immune-related genes, such as genes encoding major histocompatibility complex (MHC) II and human leukocyte antigen (HLA), compared with BM-, AD-, and PL-MSCs [145]. These immunomodulatory and immunosuppressive properties of WJ-MSCs make them more applicable for clinical use as cell therapy. A study in canines by Kang et al. revealed that canine WJ-MSCs were capable of forming new bone in recipients with bone defects after orthotopic implantation with beta tricalcium phosphate (β-TCP) for 20 weeks. The capacity of WJ-MSCs to undergo osteogenic differentiation in vitro and new bone formation in vivo was similar to that of other MSCs isolated from canine bone marrow, adipose tissue, and UCB [146]. Hence, WJ-MSCs can potentially be used in clinical bone engineering for further treatment of bone defect diseases.

### Trends in stem cell therapy for osteoporosis

The main hurdles for stem cell-based therapy for osteoporosis are long-term engraftment and the uncertainty of stem cell fate after transplantation. Certain reports have revealed that long-term engraftment of MSCs appears to be low and that the function of MSCs might be mediated through a paracrine mechanism, rather than through sustained engraftment in injured tissues [137, 147, 148]. Senescence of MSCs has been investigated as one of the key factors affecting the growth of MSCs in vitro, possibly hampering the cells’ long-term survival after transplantation [149]. Many ongoing studies are aiming to develop high-quality in vitro MSC cultures to increase the survival and engraftment rates. These developing methodologies include modification of MSCs by certain factors and improvement of in vitro MSC culture systems and differentiation procedures. The adjustment of culture conditions before transplantation, such as hypoxic preconditioning of MSC cultures in vitro, has been performed to increase the proliferation rate and to enhance the differentiation potential as well as to induce mobilization and homing of MSCs following transplantation [150, 151]. Genetically modified MSCs have been developed to ensure their homing, differentiation capacity, survival, and long-term engraftment at the injury sites of recipients. Immortalization of MSCs by knockdown of p53, a cell cycle regulator, in combination with overexpression of human telomerase reverse transcriptase (hTERT), the catalytic component of telomerase that leads to telomere elongation, could promote proliferation and increase the lifespan of MSCs while retaining the cells’ differentiation properties [152]. A combination of cell and gene therapy by overexpression of certain growth factors in MSCs has been promoted as being advantageous for MSC-based therapy [153, 154]. For example, the ectopic expression of basic fibroblast growth factor (bFGF) and platelet-derived growth factor B (PDGF-B) enhanced the in vitro proliferation and osteogenesis of BM-MSCs while inhibiting their adipogenesis [154]. MSCs are also an attractive cellular vehicle for the in vivo delivery of therapeutic genes, such as the genes encoding BMP-2 and RANK-Fc (a soluble inhibitor of RANKL), which could increase bone formation in osteoporosis animal models [155, 156]. Upon transplantation in vivo, the expressed transgene exerted its effect on both the host mesenchymal tissue (paracrine effect) and the transplanted MSCs (autocrine effect), contributing to the induction of bone formation in the recipients. These strategies of MSC modification are advantageous for the treatment of osteoporosis, which is characterized by increased bone resorption, and the therapies aim to maintain bone density and reduce the risk of fractures. To achieve effective MSC-based therapy for osteoporosis, the poor bone marrow homing and engraftment of MSCs after their systemic transplantation have to be improved. One emerging approach to overcome these limitations involves the overexpression of molecules involved in the bone homing of transplanted MSCs. Ectopic expression of α4 integrin on MSCs greatly increased bone marrow homing after systemic injection through the tail vein in immunocompetent mice. α4 integrin forms a heterodimer with endogenous β1 integrin and functions as a cell adhesion molecule, interacting with ECM proteins such as fibronectin and vascular cell adhesion protein 1 (VCAM-1) and thereby mediates the bone marrow homing and engraftment of MSCs [157]. Another study demonstrated that genetic modification of MSCs with CXCR4, the receptor for stromal-derived factor 1 (SDF-1), which mediates the bone marrow homing and engraftment of hematopoietic stem cells (HSCs), could also increase the bone marrow homing of MSCs and restore bone formation in mice with glucocorticoid-induced osteoporosis [158]. The development of in vitro differentiation procedures is quite important for MSCs used as cell therapy, especially for the treatment of localized osteoporosis and healing fractures resulting from osteoporosis. Technology consisting of three-dimensional (3D) in vitro culture models using biomaterial scaffolds has been developed, with the aim of mimicking the in vivo microenvironment to induce efficient tissue formation in vitro [159]. The biomaterial scaffolds must be slowly biodegradable and can act as a biocompatible matrix to support cell growth. In a recent preclinical study, Müller et al. demonstrated the combination of osteoconductive biomaterials with genetically modified human BM-MSCs in a bone defect rat model. The BM-MSCs were transduced with BMP-2 and loaded into β-TCP scaffolds before implantation into recipient rats. The researchers showed that when combined with BMP-2-transduced BM-MSCs, the scaffolds provided better results than scaffolds with recombinant BMP-2-treated BM-MSCs did [160]. This combination may represent a promising strategy for healing large-area bone defects in osteoporosis.

Alternative approaches involving improvement of native BM-MSCs or the local biologic environment at defect sites are of interest and are under investigation. Using a biomaterial scaffold combined with gene delivery for BMP-7 and PDGF-B expression has been shown to enhance the recruitment of BM-MSCs to defect sites and to promote their differentiation into osteoblasts, resulting in increased new bone formation in segmental femoral defects in ovariectomized rats [161]. α5β1 integrin, which mediates osteoblast differentiation in adult human MSCs through ECM-integrin interaction, is considered to be a target for promoting the osteogenic differentiation of BM-MSCs. The use of agonists that target α5β1 integrin can promote MSC recruitment and differentiation into osteoblasts and can also increase the survival of mature osteoblasts, leading to increased bone formation and repair in vivo [162]. Small molecules and microRNAs (miRNAs) are topics of interest in this area and may be applicable for osteoporosis treatment. Many miRNAs have been found to regulate the osteogenic differentiation of MSCs by various mechanisms [163]. Several miRNAs, e.g., miR-27a, miR-346, and miR-1423p, have been demonstrated to directly target inhibitors of the Wnt/β-catenin pathway, such as glycogen synthase kinase 3 beta (GSK3-β), SFRP1, and APC [164–166], resulting in modulation of the Wnt/β-catenin pathway and promotion of osteogenic differentiation of MSCs. Certain miRNAs, e.g., miR-20a, promote osteogenic differentiation by downregulating genes involved in adipogenic lineages, such as the gene encoding PPARγ [167]. By contrast, certain miRNAs negatively regulate osteogenic differentiation by targeting osteogenic genes, e.g., *RUNX2*, *OSX*, and *SATB2.* Inhibition strategy using an antagomir sequence against these miRNAs might attenuate the expression of the osteogenic genes and subsequently induce osteogenic differentiation [168–170]. The discovery of small molecules that target MSCs for fate determination by using high-throughput screening (HTS) techniques provides an advantage in drug development for osteoporosis treatment [171]. Small molecules may directly stimulate signaling pathways or target genes involved in osteogenic differentiation of MSCs [172–174]. For example, simvastatin, a 3-hydroxy-3-methylglutaryl coenzyme A (HMG-CoA) reductase inhibitor, could promote osteogenic differentiation by activating the BMP-2 pathway in an ovariectomized rat model, leading to increasing BMD and bone volume [173, 175]. Given this finding together with their other advantages, i.e., their small size, high stability, non-immunogenicity, and, most of all, cell permeability, small molecules have undoubted potential for treating osteoporosis.

## Conclusion

Osteoporosis is a systemic bone disorder defined by low BMD occurring due to an imbalance of osteoclastic and/or osteoblastic activities. The current therapeutics for osteoporosis are based on medicine for the prevention of further bone loss. The serious side effects caused by prolonged treatment have led to a need for an alternative approach for the life-long treatment of osteoporosis. Cell therapy appears to fulfill this demand, and MSCs provide a promising source of cells for clinical application in the treatment of osteoporosis. MSCs have been widely used for osteoporosis research as well as in other bone diseases due to not only their intrinsic ability to differentiate into osteoblasts but also their availability and ease of isolation, with high cell yields, from various tissues. Moreover, the immunoprivileged and immunosuppressive properties of MSCs make them more applicable in allogeneic cell replacement therapy. To date, over 400 clinical trials of MSC therapies have been registered with ClinicalTrials.gov (http://www.clinicaltrials.gov/); these trials have involved many diseases and conditions, such as bone disorders (osteoarthritis, osteogenesis imperfecta, osteoporosis, and rheumatoid arthritis), diabetes mellitus, graft-versus-host disease, and spinal cord injury. However, many questions remain unanswered, and many features have to be validated, such as the long-term engraftment and senescence of MSCs and suitable sources of MSCs for transplantation. In conclusion, much more work is needed to clarify the clinical applications of MSCs; however, the evidence certainly indicates that MSCs will play an important role in cell therapy for osteoporosis in the near future.

## Abbreviations

3D, three-dimensional; AD, adipose tissue-derived; Alox15, arachidonate 15-lipoxygenase; bFGF, basic fibroblast growth factor; BM, bone marrow-derived; BMD, bone mineral density; BMP, bone morphogenetic protein; BMPR, bone morphogenetic protein receptor; CD, cluster of differentiation; COL1A1, collagen type 1 alpha1; CSF1, colony-stimulating factor 1; CXCR4, chemokine (c-x-c motif) receptor type 4; DKK-1, dickkopf-1; Dlx5, distal-less homeobox 5; DXA, dual-energy x-ray absorptiometry; ES, embryonic stem; ESR1, estrogen receptor 1; GREM, gremlin; GSK3β, glycogen synthase kinase 3 beta; GWAS, genome-wide association studies; HLA, human leukocyte antigen; HSC, hematopoietic stem cell; iPS, induced pluripotent stem; LRP5, lipoprotein receptor-related protein 5; MARK3, microtubule affinity regulation kinase 3; MHC, major histocompatibility complex; MSC, mesenchymal stem cell; NOG, noggin; OPG, osteoprotegerin; OSX, osterix; OVX, ovariectomy; PDGF-B, platelet-derived growth factor B; PL, placenta-derived; PPARγ, peroxisome proliferator-activated receptor gamma; PTH1R, parathyroid hormone 1 receptor; RANKL, receptor activator of nuclear factor kappa B ligand; RUNX2, runt-related transcription factor 2; SDF-1, stromal-derived factor-1; SERM, selective estrogen receptor modulator; SNPs, single nucleotide polymorphisms; SOST, sclerostin; SP7, Sp7 transcription factor 7; TCF/LEF, T cell factor/lymphoid enhancer factor; TGF-β, transforming growth factor beta; TNFRSF, tumor necrosis factor receptor superfamily; UC, umbilical cord-derived; UCB, umbilical cord blood; VDR, vitamin D receptor; WHO, World Health Organization; WJ, Wharton’s jelly-derived; Wnt, wingless-type MMTV integration site family; β-TCP, beta tricalcium phosphate.
